# Geographic, demographic and economic burden of human cystic echinococcosis in Italy based on hospital discharge records (2015–2022)

**DOI:** 10.1371/journal.pntd.0014142

**Published:** 2026-03-20

**Authors:** Piero Bonelli, Giovanni Stegel, Scilla Mastrandrea, Gabriella Masu, Angela Peruzzu, Cinzia Santucciu, Giovanna Masala

**Affiliations:** 1 National Reference Laboratory of Echinococcosis (CeNRE), WOAH Reference Laboratory for Echinococcosis, Istituto Zooprofilattico Sperimentale della Sardegna, Sassari, Italy; 2 Department of Chemical, Physical, Mathematical and Natural Sciences, University of Sassari, Sassari, Italy; 3 Clinic for Infectious and Tropical Diseases, University Hospital, Sassari, Italy; Istituto Superiore Di Sanita, ITALY

## Abstract

Cystic echinococcosis (CE) is a neglected zoonotic disease that remains a public-health concern worldwide. However, poor surveillance and incomplete reporting still hinder the calculation of the CE burden. This study evaluated national hospital discharge records (HDRs) from 2015 to 2022 to update CE epidemiological situation and economic impact of human CE in Italy. We reviewed 4,293 HDRs with an echinococcosis diagnosis during 2015–2022. Data extracted included age, sex, residence, nationality, primary versus secondary diagnosis, treatment type, length of stay and discharge outcome. The average annual hospital prevalence was calculated per 100,000 inhabitants and direct hospital costs were derived from records of CE as the primary diagnosis. The 2015–2022 trend was assessed and compared to an extended data set (2001–2022). From 2015 to 2022, 2,663 cases were identified nationally, over half in the southern and insular regions. The national average annual hospital prevalence was 0.56 cases/100,000 inhabitants, peaking in the islands (1.22/100,000 inhabitants). Patients were predominantly adults (>90%) and divided into the following age categories: 25–44 (20.4%), 45–64 (30.2%) and ≥65 years (41.1%); pediatric cases were rare (<3%). The mean hospital stay for primary CE diagnosis was 10.6 days with elderly patients (>65 years) accounting for 70% of in-hospital deaths. National direct hospital costs amounted to €10,726,413, with 56% borne by the South and Islands (South 23%, Islands 33%). A downward trend in hospitalised CE cases was observed from 2015 (326 cases) to 2022 (165 cases), with a notable decline in 2020 linked to the COVID-19 pandemic. Analysis of the longer time period (2001–2022) revealed 1250 fewer cases. These findings confirm CE’s heterogeneous distribution, with persistent hyperendemic foci in traditional pastoral areas driving disproportionate healthcare costs. Sustained One Health surveillance, targeted regional prevention and robust national reporting are critical to further reduce CE burden in Italy.

## Author summary

Cystic echinococcosis (CE) is a parasitic disease transmitted mainly through a dog–sheep cycle, which can also affect humans and more than often results in a severe and costly illness. While there has been a downward trend of CE over the past two decades, the disease remains a concern, particularly in the southern and island regions where sheep farming and close contact between dogs and humans sustain transmission. Using hospital discharge data from 2015 to 2022, we estimated that there were nearly 2,700 cases of CE across the country in eight years, with the highest prevalence in Sardinia and Sicily. The disease primarily affected adults and the elderly, who had the greatest risk of experiencing complications or dying. The hospitalisation costs for CE to the Italian health system raised above €10 million between 2015 and 2022, with more than half of the financial burden falling on the endemic southern and insular regions. Although the number of cases is decreasing, CE is still underreported and requires long-term control strategies, sustained investment and political commitment. Strengthening One Health actions (disease surveillance, veterinary measures, community education) and improving national reporting will help protect people and reduce avoidable healthcare costs.

## Introduction

Cystic echinococcosis (CE) is a globally distributed zoonotic infection caused by the larval stage of *Echinococcus granulosus sensu lato* (*s.l.*), a cestode parasite belonging to the Taeniidae family. *E. granulosus s.l.* life cycle includes intermediate (wild and domestic ungulates) and definitive hosts (wild or domestic carnivores). Humans become accidental intermediate hosts through the ingestion of parasite eggs excreted in the feces of definitive hosts, typically domestic dogs and other canids. Following the ingestion of the eggs, the hexacanth larva (oncosphere) is released from the egg and penetrates the intestinal wall. The larva then migrates to the internal organs, mainly the liver and lungs, where it develops into a CE cyst (hydatid cyst) [1]. Cyst growth is typically slow, and infected individuals may remain asymptomatic for years. During this latency phase, the patient may not experience any specific symptoms until the CE cyst becomes large enough to compress adjacent organs, causing the first clinical signs. Clinical signs depend on the number and size of the CE cyst and the organ affected. Medical complications, mostly due to the cyst rupture, may occur and lead to severe disability or death, in 2% to 4% of cases [2].

CE is classified by the World Health Organization (WHO) as a neglected tropical zoonosis inflicting serious morbidity and economic loss. Globally, CE accounts for roughly 188,000 new human cases and approximately 184,000 disability-adjusted life years (DALYs) annually, at an estimated cost of about US $760 million per year [3]. Although CE has a cosmopolitan distribution, its highest endemicity occurs in areas where animal husbandry, home slaughter, and close human–dog contact facilitate transmission. Well-documented endemic regions include the Mediterranean basin, South Eastern Europe, the Middle East, Central Asia, East Africa, South America, and parts of China [4–6]. Across Europe, CE incidence varies significantly, with the highest rates observed in Italy, Spain and the Balkan countries [7,8]. Italy has long been recognized as a highly endemic focus for CE, particularly in the insular and southern mainland regions, where 90% of the entire national sheep herd is extensively reared. Despite advances in diagnostic tools and control strategies and a decreasing trend in incidences recorded in the last years, CE remains a significant public health concern in these parts of Italy [7].

While cystic echinococcosis is a notifiable parasitic infectious disease in Italy, limitations in the national health system data collection and notification reports result in underestimation and misinterpretation of the disease’s epidemiological situation, which reinforces the mistaken belief that CE poses minimal health risks. The absence of a formal surveillance system, diagnostic ambiguity and variability in regional reporting contribute to persistent challenges in estimating the true burden of the disease [7,9,10]. Despite these costraints, hospital discharge records (HDRs) are currently the only official source to estimate the CE burden in Italy. This paper aims to analyse HDRs collected from 2015 to 2022 to update epidemiological data on human CE in Italy and assess the present economic impact of the disease on the national health system.

## Materials and methods

The data used in the present study were obtained from HDRs provided by the Direzione generale della programmazione sanitaria of the Italian Ministry of Health (banca dati SDO). The HDRs, collected from all the hospitals in Italy, contained information on patients, stratified according to nationality (Italy or foreign), place of residence at NUTS 1 and NUTS 2 level (NUTS - Nomenclature of territorial units for statistics) [11], age and gender, primary or secondary diagnosis of CE, type of treatment (surgical or medical), length of stay (ordinary or day hospital) and mode of hospital discharge.

A total number of 4,293 HDRs with echinococcosis related diagnosis (primary and secondary) over an 8-year period (2015–2022) were reviewed to estimate the CE health burden. HDRs were converted into MySQL tables to allow specific data to be conveniently extracted using queries. The data exported from MySQL was then presented in aggregated form, in a spreadsheet to facilitate table and graphical representation.

HDRs were provided in anonymous form, without any patient identification code. Consequently, we were unable to distinguish hospital readmissions. To address this limitation and estimate the number of hospitalised patients affected by CE, we referred to a recent article published by Casulli et al. [7]. The study quantified the number of CE cases at a national level using various data sources and determined the corresponding number of hospitalisation admissions, finding it to be 1.6. Another previous study [9] also confirmed the accuracy of this evaluation (hospitalisations/CE cases ratio) by analysing hospital records containing a unique patient identifier, which made it possible to discriminate readmissions by the same patient. In our study, we therefore applied a correction factor of 1.6 to the number of HDRs reporting a diagnosis of echinococcosis to calculate the number of hospitalised patients affected by CE.

The average annual hospital prevalence rates were computed by counting the total number of estimated CE cases annually recorded in hospital facilities (2015–2022) and dividing them by the corresponding population of the epidemiological unit (NUTS, NUTS1 and NUTS2) per 100,000 inhabitants. General and regional statistics about Italian population per year were provided by the European Statistical System (ESS) [12].

Direct costs sustained by Italian hospital facilities for patients affected by CE in the years from 2015 to 2022, were assessed using the type of treatment received and the length of the hospitalisation, as already described [9]. As government remuneration is dependent on the primary diagnosis, only patients with a primary diagnosis of echinococcosis were considered for quantification of the direct costs. Similarly, in-hospital mortality rates were calculated only for patients with a primary diagnosis of CE.

Finally, the trend of CE in humans was analysed using data from 2015 to 2022. To extend the analysis over a longer period, we created a comprehensive dataset by integrating HDRs records from 2001 to 2014, which were available from our previous researches [9,10]. In line with the 2015–2022 period, the estimated number of human CE cases were calculated for the larger dataset by applying the correction factor. Statistical analyses were performed using one-way ANOVA, followed by Tukey’s pairwise comparison and paired T-test where appropriate. Differences were considered significant when P ≤ 0.05.

## Results

A total number of 4,293 HDRs from Italian hospitals over a period of 8 years (2015–2022) were analysed. Out of 4,293 HDRs, 1,776 (about 41%) were associated with patients who were admitted to hospital with CE as the primary diagnosis, while 2,487 (58%) were related to patients who had CE as a secondary diagnosis and were admitted for other medical conditions. Although Italy is not an endemic country for *E. multilocularis*, 32 HDRs (<1%) with a diagnosis of alveolar echinococcosis were additionally found and excluded from the database even though they were most likely misdiagnosed cystic echinococcosis.

In Table 1, are listed the number of the estimated CE cases stratified by macroregions (NUTS1), regions (NUTS2) and age groups. CE cases were found to be 2,663 throughout the country. The southern and insular regions accounted for 50.6% (n = 1,348) of the total cystic echinococcosis. The vast majority of patients (>90%) were adults, including 20.4% young adults (25–44 years), 30.2% middle aged adults (45–64 years) and 41.1% senior adults (over 65 years). Only 0.5% of CE cases were aged under 4 years and child and adolescent accounted for 2.4% of the total number of patients.

**Table 1:** The estimated number of CE cases by age category and region of residence admitted to Italian hospital facilities over eight years (2015-2022).

ITALIAN MACROREGIONS (NUTS1)	ITALIAN REGION (NUTS2)	AGE GROUP (YEARS)							
		0-4	5-14	15-24	25-44	45-64	>65	TOTAL PER REGION	TOTAL PER MACROREGION
**NORTH-WEST**	**PIEMONTE**	0	2	6	29	36	36	109	442
	**VALLE D’AOSTA**	0	0	1	0	1	1	3	
	**LOMBARDIA**	1	5	13	78	98	86	281	
	**LIGURIA**	0	1	2	12	11	23	49	
**NORTH-EAST**	**E. ROMAGNA**	1	5	19	64	63	59	211	379
	**TRENTINO (BZ)**	0	0	2	2	6	1	11	
	**TRENTINO (TN)**	0	0	2	3	2	3	10	
	**VENETO**	1	4	10	36	32	21	104	
	**FRIULI V. GIULIA**	0	1	7	19	7	9	43	
**CENTER**	**TOSCANA**	0	9	8	32	38	38	125	494
	**UMBRIA**	0	0	4	8	8	19	39	
	**MARCHE**	0	2	2	11	16	20	51	
	**LAZIO**	2	19	13	42	82	121	279	
**SOUTH**	**ABRUZZO**	1	0	8	11	19	33	72	706
	**MOLISE**	1	1	0	0	4	13	19	
	**CAMPANIA**	3	3	13	47	64	74	204	
	**PUGLIA**	2	5	17	26	60	141	251	
	**BASILICATA**	0	0	2	9	9	35	55	
	**CALABRIA**	1	4	2	22	34	42	105	
**ISLANDS**	**SICILIA**	0	2	12	48	107	153	322	642
	**SARDEGNA**	0	1	4	43	107	165	320	
	**ITALY**	13	64	147	542	804	1093	2663	2663

**Fig 1:**
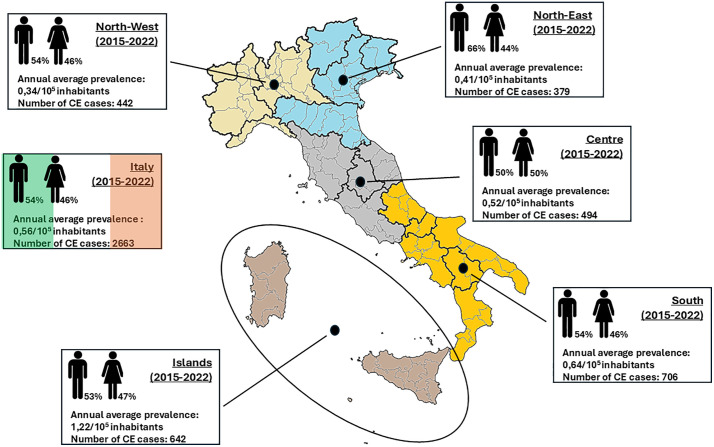
Annual average hospital prevalence and number of the estimated CE human cases in Italy from 2015 to 2022. Macroregions are defined as NUTS1 following the classification of territorial units for statistics (NUTS). The cliparts and the base layer of the map were obtained from the open source platform https://commons.wikimedia.org (direct link to cliparts: https://commons.wikimedia.org/wiki/File:Toilet.svg; direct link to the map base layer: https://commons.wikimedia.org/wiki/File:Italian_regions_white.svg; license terms: https://commons.wikimedia.org/wiki/Commons:Reusing_content_outside_Wikimedia).

The annual national hospital prevalence averaged 0.56 per 100,000 inhabitants, with the highest ratio being recorded in the islands (1.22/100,000 inhabitants; p = 0,0001). We found an equal prevalence of CE among males and females at both the national and macroregional levels. However, a slightly higher male-to-female ratio was observed in north east Italy (Fig 1).

**Fig 2:**
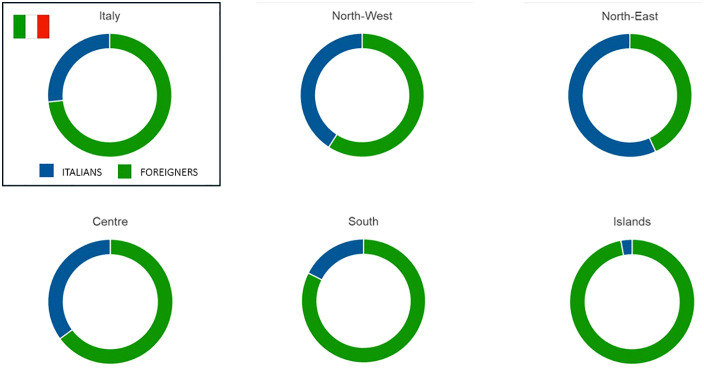
Proportion of the Italian and foreign estimated CE human patients in Italy from 2015 to 2022. Macroregions are defined as NUTS1 following the classification of territorial units for statistics (NUTS).

Fig 2 illustrates the nationality of the estimated human CE patients in Italy, reflecting the higher presence of foreigners in the northern regions (North West: 41%; North East: 57%), compared to the Centre (35%), the South (18%), and the Islands (3%).

The length of hospital stays and in-hospital mortality for patients with primary diagnosis of CE by age category in Italian hospital facilities from 2015 to 2022 are reported in Table 2. Irrespective of age category, CE patients admitted to hospital for CE spent an average stay of approximately 10 days. Patients over the age of 65 accounted for 70% of the mortalities.

**Table 2:** Hospital stays, expressed as first (25^th^ percentile), second (median) and third quartile (75^th^ percentile) and in-hospital mortality of patients with CE primary diagnosis by age category in Italian hospital facilities (2015-2022).

	LENGHT OF HOSPITAL STAY (25^th^-75^th^percentiles)	IN-HOSPITAL MORTALITY (Number/total)
**0-4 years**	5 (3–8.5)	0/13
**5-14 years**	4 (2–9)	0/64
**15-24 years**	7 (4–12)	0/147
**25-44 years**	8 (4–12)	1/542
**45-64 years**	7 (4–11)	4/804
**>65 years**	8 (5–15)	12/1093
**All patients**	7 (4–12)	17/2663

**Fig 3:**
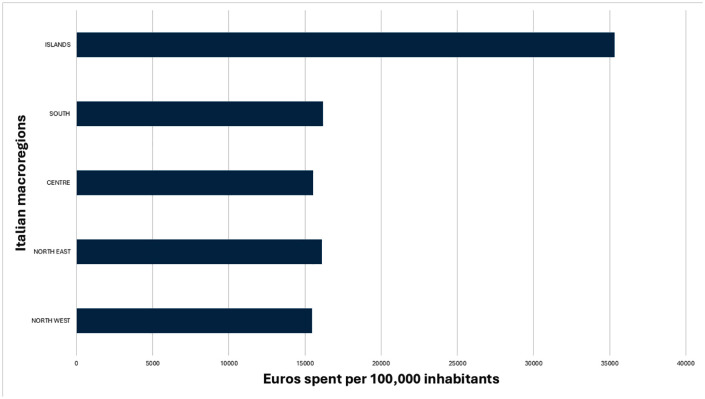
Direct costs associated to human CE: average amount of euros spent per 100.000 inhabitants in Italy’s macroregions over the years from 2015 to 2022.

Overall direct costs associated with human CE, provided by the national health services, to hospitals during the year 2015–2022 are illustrated in Table 3. To better assess the economic impact of CE on the macro-regions, we evaluated the direct costs per inhabitants (Fig 3). The Italian healthcare system incurred direct costs of € 10,726,413 for the hospitalisation of CE patients, of which € 7,862,045 related to surgical treatments (73%) and € 2,864,369 to medical treatments (27%) (Table 4). The proportions of surgical and medical treatments in patients hospitalised for CE remained stable during the study period, except for the year 2022, when surgery rates increased compared to medical therapy. The average Italian annual economic burden of CE in 2015–2022 was € 2,243 (SD = 559; SEM = 198) per 100,000 inhabitants.

**Table 3:** Total direct costs associated with human CE sustained by hospital facilities in Italy over the years from 2015 to 2022 differentiated by NUTS1 and NUTS2.

ITALIAN MACROREGIONS (NUTS1)	ITALIAN REGION (NUTS2)	2015 (€)	2016 (€)	2017 (€)	2018 (€)	2019 (€)	2020 (€)	2021 (€)	2022 (€)	2015-2022 (€)
**NORTH-WEST**	**PIEMONTE**	53,520	67,465	122,822	77,123	97,805	72,412	27,890	50,410	569,452
	**VALLE D’AOSTA**	0	0	6,972	3,109	0	3,109	0	0	13,192
	**LOMBARDIA**	284,562	255,823	235,471	210,123	219,168	119,384	129,984	242,467	1,696,981
	**LIGURIA**	20,823	43,768	24,028	40,329	6,219	659	36,795	17,055	189,675
**NORTH-EAST**	**E. ROMAGNA**	184,867	137,427	171,537	146,097	94,508	69,208	87,630	137,468	1,028,741
	**TRENTINO (BZ)**	0	3,439	6,219	3,109	6,973	10,082	0	0	29,822
	**TRENTINO (TN)**	6.973	0	6,219	13,192	6,219	0	0	3,109	35,712
	**VENETO**	62,330	120,986	84.850	124,001	58,042	54,274	44,192	68,973	617,648
	**FRIULI V. GIULIA**	34,909	40,752	21,812	25,136	20,164	3,439	659	11,071	157,941
**CENTRE**	**TOSCANA**	86,782	55,451	68,784	79,809	58,467	37,549	63,932	32,648	483,420
	**UMBRIA**	6,973	27,137	28,220	29,493	10,082	10,082	23,603	20,918	156,508
	**MARCHE**	23,274	22,850	6,219	16,301	53,190	20,918	13,945	17,055	173,752
	**LAZIO**	142,798	196,788	155,325	95,921	159,523	95,686	94,603	87,630	1,028,272
**SOUTH**	**ABRUZZO**	34,344	232,74	40,329	58,117	38,302	19,411	23,274	21,577	258,627
	**MOLISE**	10,082	0	0	6,973	6,973	3,109	6,973	0	34,110
	**CAMPANIA**	102,987	188,262	114,013	67,795	66,193	78,536	68,784	62,189	748,759
	**PUGLIA**	109,631	77,877	107,934	149,960	98,466	77,548	67,466	81,740	770,622
	**BASILICATA**	33,685	10,412	23,179	10,082	10,412	30,152	16,631	0	134,552
	**CALABRIA**	60,349	48,949	66,428	21,907	3,810	14,839	17,055	17,290	286,625
**ISLANDS**	**SICILIA**	231,041	209,839	253,796	94,178	142,987	89,043	112,175	107,275	1,240,336
	**SARDEGNA**	174,222	240,979	106,098	122,728	130,030	63,932	106,427	127,250	1,071,667
	**ITALY**	1,664,152	1,771,477	1,650,254	1,395,482	1,323,533	873,372	942,017	1,106,126	10,726,413

**Table 4:** Total direct costs associated with human CE sustained by hospital facilities in Italy over the years from 2015 to 2022 differentiated by surgical and medical treatments.

		2015	2016	2017	2018	2019	2020	2021	2022	2015-2022
**ITALY**	**Total direct costs in euros**	**1,664,152**	**1,771,477**	**1,650,254**	**1,395,482**	**1,323,533**	**873,372**	**942,017**	**1,106,126**	**10,726,413**
	Surgical treatment (%)	1,160,807 (70%)	1,321,323 (75%)	1,192,419 (72%)	960,587 (69%)	965,716 (73%)	648,459 (74%)	700,754 (74%)	911,979 (82%)	7,862,045 (73%)
	Medical treatment (%)	503,345 (30%)	450,154 (25%)	457,835 (28%)	434,895 (31%)	357,817 (27%)	224,912 (26%)	241,263 (26%)	194,147 (18%)	2,864,368 (27%)

**Fig 4:**
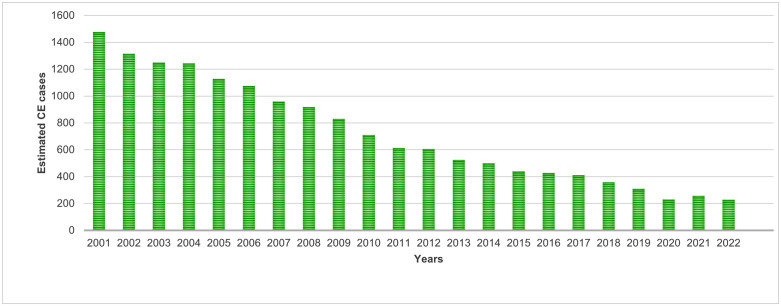
CE trend in Italian hospital facilities from 2001 to 2022.

During the period covered in this study, CE trend, in Italy, (Fig 4) appeared to be decreasing from 2015 to 2022. The number of Italian patients decreased gradually and steadily from 326 in 2015–165 in 2022. However, there was an abrupt drop in 2020 when the national health system’s focus was almost entirely on solving the emergency caused by the COVID-19 pandemic. Similarly, data recorded for foreign patients, who accounted for between 26% and 29% of the total, showed a similar downward pattern from 2015 (114 CE cases) to 2022 (63 CE cases), with a clear decrease in 2020 due to the pandemic. Considering the larger dataset of HDRs that was built by adding previous records collected from CeNRE (2001–2014) [9,10], it is clear that the decrease in human CE cases was significantly more pronounced, as some 1250 fewer cases were reported between 2001 and 2022.

## Discussion

This study provides an updated national overview of the epidemiological and economic burden of the human cystic echinococcosis in Italy, spanning an 8-year period from 2015 to 2022. The analysis of 4,293 hospital discharge records (HDRs) highlights important geographical, demographic, and economic aspects of the disease that remain relevant despite the overall decreasing trend in CE hospitalisations reported in Italy.

The inadequate and unreliable recording of human CE cases in Italy has led us to use information from HDRs, which currently represent the only official data available. There are various limitations to this approach, since asymptomatic patients may be diagnosed accidentally or in an outpatient setting, or even may not require hospitalisation, thus *de facto* eluding the HDRs reporting system. The latest European Union One Health 2024 Zoonoses Report clearly showed that Italy did not notify any cases of human echinococcosis from 2020 to 2022, only reporting 15 and 17 cases, in 2023 and 2024 respectively, without distinction between Echinococcus species [13]. Our findings revealed 716 human CE cases from 2020 to 2022, indicating a clear discrepancy with consistent underestimation of the burden of CE in Italian official data. Beyond underreporting, CE may be confused with other conditions sharing similar morphological characteristics, as it occurs particularly, in some liver cystic and solid lesions which are more likely to be misdiagnosed [14,15]. To a lesser extent, there is a possibility that CE might be recorded as alveolar echinococcosis. Notably, the first, and to date the only locally acquired case of infection with *Echinococcus multilocularis,* was reported in 2023 [16]. During the 8-year study period, 32 HDRs were attributed to alveolar echinococcosis, highlighting the potential for misdiagnosis. This points out the significance of precise documentation and the opportunity to further refine the differential diagnosis process, thereby optimising the efficacy of national reporting systems.

Consistent with previous reports [9,10], our results reinforce the heterogeneous distribution of CE across the Italian territory. Although the annual national average hospital prevalence remains relatively low (0.56/10^5^ inhabitants), significant regional variation can still be seen, with the highest prevalence reported in the insular regions (1.22/10^5^ inhabitants), particularly in Sardinia (2.47/10^5^ inhabitants).

These findings highlight the persistence of hyperendemic foci in certain macroregions, which reflected socio-economic and agricultural factors, particularly in areas where extensive sheep farming is widely practised [17]. As already reported, CE is more likely to be acquired by people living in rural localities who engage in agricultural activities and livestock management [18,19]. Previous researches showed that environmental contamination is one of the main risk factors for the CE transmission, primarily through routes of infection involving hand-to-mouth contact, while consumption of contaminated food and water may be of secondary importance [20–22]. Although the relative importance of these pathways of infection remains to be fully understood, it may vary depending on the prevalence rates in specific geographic areas and sociocultural habits [23]. The significance of the sheep-dog cycle in the transmission patterns of the parasite is well established, ensuring the perpetuation of the life cycle through at-risk practices including illegal slaughter, feeding dogs with raw viscera and unappropriated carcasses disposal.

As already described [5], we observed a lower prevalence of CE in the northern regions of Italy, particularly in the North West (0.34/10^5^ inhabitants), that may suggest a less effective *E. granulosus* life cycle perpetuation as well as a higher migratory pressure in these areas. This observation should also consider the possibility that foreign-born patients may have acquired the infection in their country of origin. Indeed, a higher proportion of CE patients of foreign nationality are living in the northern regions of Italy (52% Italians, 48% foreigners). The North East, in particular, represents a better economic opportunities and a long-term settlement for foreign populations [24,25].

From a demographic perspective, and in line with other researches [9,26,27], more than 90% of patients belonged to adult age groups, with a concentration in both the middle and older age classes. This pattern reflects the long incubation period, the low mortality rate of the disease and the slow development of CE cysts before clinical manifestation. The higher mortality observed in elderly patients (>65 years), who accounted for 70% of in-hospital deaths, underscores the impact of comorbidity and the clinical vulnerability of this population. On the other hand, pediatric cases were rare (<3%), suggesting limited ongoing transmission in younger cohorts. Nevertheless, children have been observed to present severe clinical forms of the disease (e.g., cerebral CE cyst localization) more frequently than the other age groups. This must be considered when assessing the total burden of CE [28,29].

The sex distribution shows a similar prevalence between males and females, consistent with reports at the European level [7]. However, data from the Global Burden of Disease Study 2019 indicated a higher global disease burden in women compared to men [26,27]. The slight male predominance in the North East might reflect occupational or socio-cultural factors influencing exposure risks.

Over the study period, the national health system incurred direct hospitalisation costs exceeding €10.7 million, with more than half the expenditure and CE cases concentrated in the southern and insular Italian regions, where traditional pastoral and livestock-rearing practices are still prevalent. When adjusted to the population size, these areas still bore a disproportionate share of the financial burden. A potential targeted regional investment in prevention and long-term control efforts, including public health education, dog deworming programs and sheep vaccination may prove cost-effective as shown in other endemic settings [30–33].

Importantly, on one hand the observed downward trend in costs was primarily driven by a steady decrease in the hospitalisation numbers, rather than by any significant changes in treatment type (surgical or medical) that could account for the reduction in expenses. On the other hand, despite the increase of reimbursements by the Italian National Health Service in 2013, the average annual economic burden of CE decreased by almost 3-fold, from €6,398 per 100,000 inhabitants (2001–2014) to €2,243 per 100,000 inhabitants (2015–2022) [9].

Our results are consistent with the broader European epidemiological context. Casulli et al. [7], reported decreasing CE trends in historically endemic Mediterranean countries, including Italy, and identified southeastern Europe as the current European hotspot. Conversely, Yang et al. [26] showed that the global burden of CE has stayed high over the past 30 years, particularly in Central Asia, North Africa, and the Middle East.

The abrupt decline in 2020 coincided with the COVID-19 pandemic, when routine healthcare access was disrupted. This phenomenon has also been noted in other neglected diseases, where diagnosis and treatment were deprioritized during the health emergency, mainly in middle and low income countries [34]. The rebound to pre-pandemic trends in subsequent years indicates that the decline was artificial rather than epidemiological.

Overall, our findings indicated that CE in Italy is following a long-term decreasing trend, in both prevalence and economic impact. Nevertheless, it remains a significant health concern, particularly in the southern and insular regions of Italy. Continued investment in One Health-based surveillance, veterinary interventions on definite and intermediate hosts and health education are paramount to consolidate the progress achieved and to prevent disease persistence in vulnerable communities. But, given the underreporting issues, our data likely underestimate the true burden, reinforcing the need to strengthen national reporting systems in line with the WHO 2021–2030 roadmap for neglected tropical diseases [35]. As highlighted by previous experiences, the success of control programs depends on the steady economic sustainability, political commitment and culture tailored interventions [36,37]. The recent report of the re-emergence of CE after ending the *E. granulosus* control program in Chile provides us with a valuable lesson for the control of zoonotic diseases, which points out the importance of sustained efforts and community engagement [38].
